# The Effect of Lattice Misfits on the Precipitation at Dislocations: Phase-Field Crystal Simulation

**DOI:** 10.3390/ma16186307

**Published:** 2023-09-20

**Authors:** Hong Mao, Changlin Zeng, Zhikang Zhang, Xiong Shuai, Sai Tang

**Affiliations:** 1 Hunan Institute of Science and Technology, College of Mechanical Engineering, Yueyang 414006, China; paomajun@163.com (H.M.); 822211110509@vip.hnist.edu.cn (C.Z.); 2719zryjx223@163.com (Z.Z.); 2 School of Chemical Engineering and Technology, Xi’an Jiaotong University, Xi’an 710049, China; 3 International Institute for Inovation, Jiangxi University of Science and Technology, Nanchang 330013, China; 4 State Key Lab for Powder Metallurgy, Central South University, Changsha 410083, China

**Keywords:** lattice misfit, dislocation, precipitation, phase-field crystal simulation

## Abstract

An atomic-scale approach was employed to simulate the formation of precipitates with different lattice misfits in the early stages of the aging of supersaturated aluminum alloys. The simulation results revealed that the increase in lattice misfits could significantly promote the nucleation rate of precipitates, which results in a larger number and smaller size of the precipitates. The morphologies of the precipitates also vary with the degree of a lattice misfit. Moreover, the higher the lattice misfit, the earlier the nucleation of the second phase occurs, which can substantially inhibit the movement of dislocations. The research on the lattice misfit of precipitation can provide theoretical guidance for the design of high-strength aluminum alloys.

## 1. Introduction

Dislocations, as common crystal defects, are widely present in metals. Compared to perfect crystals, the regions around dislocations are more favorable for the adsorption of solute atoms, thereby inducing precipitation [1,2]. The precipitation caused via dislocations in supersaturated alloys has a significant impact on the microstructure and mechanical properties of alloys [3]. Dislocation-induced precipitation can lead to a pinning effect, through which dislocations are pinned by the precipitate phase, hindering their slip motion. This pinning effect can significantly enhance the strength, hardness, and deformation resistance of the alloy [4]. To control the size and morphology of dislocation-induced precipitation, various methods, such as adjusting the alloy composition, heat treatment conditions, and applied stress, can be employed. Precise control over the process of dislocation-induced precipitation can result in the formation of high-density nano-sized precipitates. These nano-sized precipitates act as dispersion strengtheners in the alloy, impeding the slip of crystals and the motion of dislocations, thus significantly improving the mechanical properties of the alloy [1,5]. The lattice mismatch between the matrix phase and the precipitate phase is a crucial factor influencing the process of dislocation-induced precipitation. When there is a lattice mismatch between the matrix and precipitate phases, it induces coherent elastic strains. These strains can affect the microstructure of the precipitates, such as by altering their grain size, shape, and orientation. The lattice mismatch can be controlled by adjusting the alloy composition and heat treatment conditions, enabling the control of the microstructure of the precipitates [6,7]. In conclusion, dislocations are common crystal defects in metals, and dislocation-induced precipitation plays a significant role in the microstructure and mechanical properties of metal alloys. By controlling the size and morphology of dislocation-induced precipitation and adjusting the lattice mismatch, precise control over the microstructure of the alloy can be achieved, leading to significant improvements in its performance.

It has been reported in recent research that the popularity and size of precipitates could be manipulated via tuning the magnitude of the lattice misfit between the matrix and precipitated phases to enhance the mechanical properties of the alloy [8,9,10]. The lattice misfit not only determines the structure and stress state of the two-phase interface but also has an assignable influence on the microstructure and mechanical properties of alloys. Nickel-based alloys are widely used in high-temperature and high-strength environments. One of the common precipitates is the γ′ phase. The formation of the γ′ phase can be achieved by adding appropriate alloying elements, such as aluminum and titanium. When the lattice mismatch between the γ′ phase in the nickel-based alloy and the matrix is small, the lattice mismatch is less than 1%, the compatibility between the γ′ phase and the matrix is higher. In this case, the γ′ phase usually precipitates in a high-density form, forming uniform nanoparticles distributed in the matrix. These nanoparticles can effectively hinder the movement of dislocations and the slip of grain boundaries, thereby significantly improving the strength and deformation resistance of the alloy. On the contrary, when the lattice mismatch is large, the lattice mismatch is greater than 1%, the compatibility between the γ′ phase and the matrix is low. In this case, the density number of the γ′ phase may decrease, and the size may increase. Such a lattice mismatch will lead to an increase in interface stress, which will promote the growth and coalescence of the γ′ phase and form a larger precipitate. These larger precipitates may lead to a decrease in the mechanical properties of the alloy because they may become the source of dislocations and the starting point of cracks. Therefore, by adjusting the lattice mismatch between the matrix and the precipitated phase in a nickel-based alloy, the microstructure of the alloy can be significantly affected. Reasonable control of lattice mismatches can achieve a high density and uniform distribution of nano-precipitates, thereby improving the mechanical properties of the alloy. For example, Jiang et al. obtained high-density nano-precipitates and high shear stress by minimizing the lattice mismatch. Through this measure, the nucleation potential barrier of intermetallic compound particles is significantly reduced, promoting the uniform dispersion distribution of smaller-scale (2–5 nm) nanoparticles and significantly improving the volume density and thermal stability of strengthened particles. At the same time, the low mismatch coherent interface, combined with a small scale, effectively alleviates the micro-elastic deformation around the enhanced particles, improving the material’s macroscopic uniform plastic deformation ability; on the other hand, the introduction of the “chemical ordering effect” as the main strengthening mechanism effectively hinders the shear effect of dislocations on the reinforcing phase particles, thereby significantly improving the mechanical properties of an alloy [11]. In addition, this is particularly significant for precipitation-strengthening alloys such as aluminum alloys because such alloys are strengthened through a wide variety of precipitated phases. The coherency, number density, size, and precipitation behavior of the precipitate–matrix interface may be different and deeply depend on the lattice mismatch [10,12,13,14,15,16].

However, due to the limitations of experimental conditions, these studies have remained at a preliminary qualitative stage, and our understanding of the micro-mechanisms of lattice mismatches on precipitation and precipitation processes is limited [17]. Firstly, changing the lattice mismatch in experimental studies often requires the addition of some auxiliary substances, which inevitably introduces unpredictable errors and is not conducive to studying the influence of lattice mismatches on the morphology of precipitation phases. In addition, observing the interaction between dislocations and precipitation phases in experiments is challenging. To overcome the limitations of experiments, numerical simulations have become a powerful tool for studying the micro-mechanisms of lattice mismatches in precipitation and precipitation processes. Many phase-field simulations have been used to reveal the evolution and kinetics of precipitation morphology under different lattice mismatch sizes [18,19,20]. These simulation methods simulate the evolution of crystal structures by describing the phase-field variables in the material. However, phase-field methods have some limitations in observing the micro-mechanisms of precipitation processes, as they blur the atomic-scale details of the solid phase. Another commonly used simulation method is molecular dynamics, which can simulate atomic-scale motion and interactions. However, the simulation time scale of molecular dynamics limits our ability to observe complete precipitation processes [21]. To reveal the micro-mechanisms of precipitation processes, we need a method that can describe atomic-scale precipitation processes on diffusion time scales. The phase-field crystal model is an ideal choice. In the past two decades, the phase-field crystal model has been successfully used to simulate the microstructural evolution phenomena of various materials, including nucleation [22], crystal growth [23], grain growth, defect dynamics [24], and precipitation [25]. The phase-field crystal model can simulate the phase-field variables in a material, and the evolution of these variables can be determined by minimizing the free energy function. By adjusting the parameters of the free energy function, we can simulate the characteristics and behaviors of different materials. In the precipitation process, the phase-field crystal model can describe the diffusion and interactions of atoms. It can simulate the motion and phase transformation of atoms in a material, thus revealing the micro-mechanisms of precipitation processes. Through this model, we can observe the aggregation and arrangement of atoms, as well as the formation and evolution process of precipitation phases. By studying the phase-field crystal model, we can gain a deeper understanding of the influence of lattice mismatches on precipitation and precipitation processes. These models can describe diffusion and interactions in precipitation processes at the atomic scale, thus revealing micro-mechanisms. This is of great significance for alloy design and material performance improvement in the fields of materials science and engineering. Although there are limitations in experimental conditions, through numerical simulations, we can better understand the influence of lattice mismatches on precipitation and precipitation processes.

In this paper, we used the structure phase-field crystal (SPFC) model, as the most recent PFC formalism, to perform atomistic simulations of dislocation-induced precipitation in samples with various lattice misfits. Our work aimed to (1) determine how lattice misfits affect the micro-mechanism during dislocation-induced precipitation and (2) analyze the effect of lattice misfits on the role of dislocation in nucleation and the growth of precipitates. We found that a high lattice misfit can lower the nucleation energy barrier for precipitation, making it easier to form new nano-precipitates. This leads to an increase in the number density of nano-precipitates and a reduction in their size. This is of significant importance for improving the properties of materials and controlling nanostructures. Additionally, our study revealed the role of lattice misfits in the precipitation and growth processes of dislocations. We observed that a high lattice misfit can accelerate the rate of dislocation-induced precipitation, thereby hindering the movement of dislocations within the material. This implies that lattice misfits can influence the mechanical properties and deformation behavior of materials by affecting the motion of dislocations. Through these research findings, we can better understand the micro-mechanisms of dislocation-induced precipitation influenced by lattice misfits. This is of great significance for materials design and performance improvement, especially in the fields of alloys and nanomaterials. Our study provides a foundation for a further investigation into the impact of lattice misfits on material properties and serves as a valuable reference for materials engineering.

## 2. Methods

### 2.1. SPFC Model and Simulations

The SPFC model approximates the classical density functional theory (CDFT) of freezing, simplifying the calculation by fitting the two-point correlation function in the CDFT with the Gaussian function. The free energy functional is included in the binary SPFC model [26].

(1) $$\frac{\Delta F}{kT\rho^{o}}=\int fdr=\int \{\frac{n^{2}}{2}-\eta \frac{n^{3}}{6}+\chi \frac{n^{4}}{12}+(n+1)\Delta F_{mix}-\frac{1}{2}n{\int dr^{’}C_{eff}^{n}(|r-r^{’}|)n^{’}+\alpha |\vec{\nabla }c|}^{2}\}dr$$

Here, *n* and *c* are the rescaled particle number density field and the solute concentration fields, respectively. *η* = *χ* = 1 describes a Taylor-series expansion of the bulk free energy around the reference density, and $\Delta F_{mix}$ denotes the entropy of mixing:

(2) $$\Delta F_{mix}=\omega \{c\ln(\frac{c}{c_{o}})+(1-c)\ln(\frac{1-c}{1-c_{o}})\}$$

The coefficient *ω* is a fitting parameter to fit the entropic energy away from the reference composition *c*_0_. The parameter α is a coefficient considered the energy of compositional interfaces (assume α equals 1). These parameters are discussed further in Ref. [25].

(3) $$C_{eff}^{n}=X_{1}(c)C_{2}^{AA}+X_{2}(c)C_{2}^{BB}$$

where

*X*_1_(c) = 1 − 3*c*^2^ + 2*c*^3^; (4)

*X*_2_(*c*) = 1 − 3(1 − *c*)^2^ + 2(1 − *c*)^3^ (5)

are interpolation functions associated with the two correlation functions $C_{2}^{AA}$ and $C_{2}^{BB}$, which contribute to the excess free energy for the cases in which A atoms are in a crystalline structure preferred by B atoms, and B atoms are in a structure preferred by A atoms. The correlation functions ${\hat{C}}_{2}^{ii}$ is defined as reciprocal space peaks at positions determined by each component’s equilibrium crystal unit structure. Each peak is represented by the following Gaussian form of width α*_j_*, modulated for temperature via a Debye–Waller prefactor accounting for a sufficient transition temperature σ*_Mj_*.

(6) $${\hat{C}}_{2j}^{ii}=e^{-\frac{\sigma^{2}}{\sigma_{Mj}^{2}}}e^{-\frac{(k-k_{j}{)}^{2}}{2\sigma_{j}^{2}}}$$

where *ii* = *AA*, *BB*. The total kernel ${\overset{ˇ}{C}}_{2}^{ii}$ is taken as the envelope of all peaks ${\overset{ˇ}{C}}_{2}^{ii}$ included to represent the atomic interactions. The total density is defined as the accumulation of the density of each component ($\rho =\rho_{A}+\rho_{B}$, $\rho^{o}=\rho_{A}^{o}+\rho_{B}^{o}$). The equation of motion can be written as

(7) $$\frac{\partial n}{\partial t}=\vec{\nabla }\cdot \{M_{n}\vec{\nabla }(\frac{\delta F}{\delta n})\}+\eta_{n}(\sigma ,t)$$

(8) $$\frac{\partial c}{\partial t}=\vec{\nabla }\cdot \{M_{c}\vec{\nabla }(\frac{\delta F}{\delta c})\}+\eta_{c}(\sigma ,t)$$

$M_{n}$ and $M_{c}$ are dimensionless kinetic mobility parameters (assumed to be equal to 1). $\eta_{n}\left(\sigma ,t\right)$ and $\eta_{c}\left(\sigma ,t\right)$ represent the Gaussian noise response to the effect of fast atomic vibrations in density and concentration fields, respectively. Here, *M_n_* and *M_c_* are 0.1 and 10, respectively.

### 2.2. Simulation Detail

This work aimed to investigate the effect of lattice misfits on dislocation-induced precipitation by counting the evolution process of a large number of dislocation precipitates on various alloy samples with different lattice misfits. Thus, the initial condition is a supersaturated solid solution distorted by introducing a uniform distribution of partial edge dislocations with a number density of ≅1 dislocation per 200 atoms [27]. The initial concentration was *c*_0_.

The lattice misfit is defined as $f={(a}_{0}-a_{1})/a_{0}$, where $a_{0}$ and $a_{1}$ are the lattice constants of the matrix and precipitates, respectively. As we know, aluminum alloys are characterized by diversified precipitates, and the lattice misfits between the matrix and precipitates range from small (Al_3_Zr, 0.27%) to large (AlLi, 21.4%) [10,12,28]. To pursue generality in this study, we investigated the precipitation processes of precipitates with magnitudes of lattice misfits spanning from 7.5% to 20%, rather than a unique precipitate. Thus, the dependence of precipitation behaviors on lattice misfits could be revealed.

The simulation was performed on a 2D rectangular mesh with grid spacing dx = 0.125 and time step dt = 0.5. The simulation domain consisted of 3840 × 3840 grid spacings (equivalent to 480 × 480 atoms). The lattice parameter of 1 was set for the matrix phase, and each lattice spacing was divided into 8 mesh spacings. A semi-implicit Fourier-spectral method was used to solve the dynamic equations [29].

## 3. Results and Discussion

### 3.1. Precipitation Simulations

**Figure 1:**
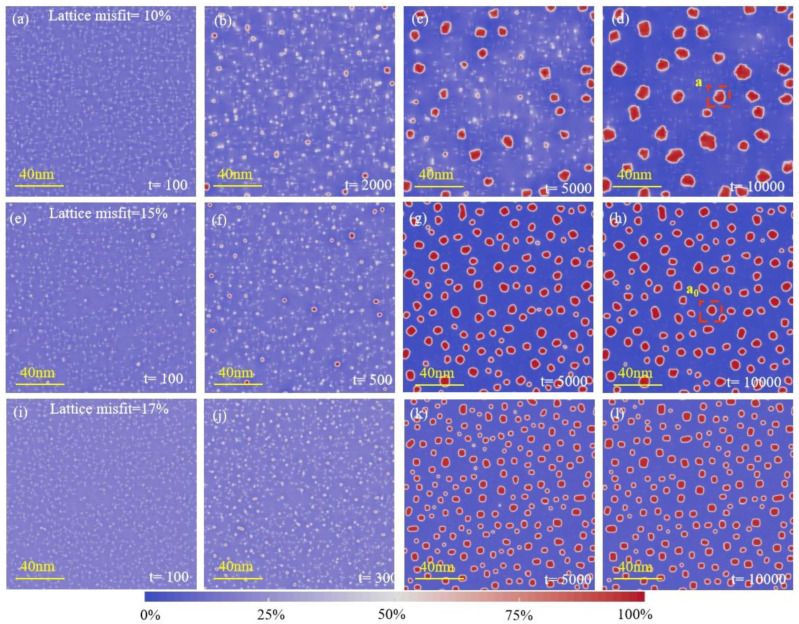
Dislocation-induced precipitation. Relatively low lattice misfit (10%) alloy (the color code means the concentration of the precipitation phase): (**a**) t = 100; (**b**) t = 2000; (**c**) t = 5000; (**d**) t = 10,000; relatively high lattice misfit (15%) alloy: (**e**) t = 100; (**f**) t = 500; (**g**) t = 5000; (**h**) t = 10,000. The higher-lattice-misfit (17%) alloy: (**i**) t = 100; (**j**) t = 300; (**k**) t = 5000; (**l**) t = 10,000.

**Figure 2:**
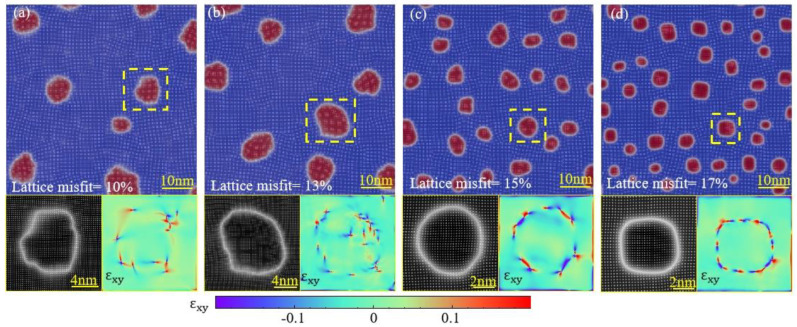
Morphology of precipitates and elastic field under various lattice misfits: (**a**) f = 10%; (**b**) f = 13%; (**c**) f = 15%; (**d**) f = 17%. (t = 10,000).

Figure 1 shows the PFC simulation results for dislocation-induced precipitation’s concentration evolution and morphology evolution on three kinds of lattice misfits: 10%, 15%, and 17%. Labeled on these images are the typical stable clusters “*a*” and “*a*_0_” in the alloys with different lattice misfits that survived the growing competition among the other clusters. As can be inferred from Figure 1, the number of observed clusters within the unit area of simulation at each time step is much more significant for the alloy with a higher lattice misfit. Clusters can be observed in the alloy with a 15% or 17% lattice misfit before t = 500 but not in the alloy with a lower lattice misfit (10%) until t = 2000, which means that the increasing lattice misfit can reduce the nucleation incubation time of clusters. The zoomed-in images of the area within the box labeled cluster “*a*” (in Figure 1) are shown in Figure 2.

Figure 2 shows the microstructure of the precipitates of the alloys after the same aging (t = 10,000). Figure 1 and Figure 2 demonstrate that the increase in a lattice misfit causes a change from irregular to spherical precipitate morphology, while a higher lattice misfit causes a change from spherical to cuboidal precipitate morphology. Ferreiros observed a similar morphological change of cuboidal L_12_ precipitates in alloys of the Fe-Al-Ti system [30,31]. They considered it a consequence of the increase in the lattice misfit between the matrix and the precipitate in the alloy containing Ti [32]. In our simulation, this phenomenon can be further attributed to the role of the misfit strain at the boundary of precipitates. As shown in Figure 2, the incoherency increases with the magnitude of the lattice misfit. The lattice misfit between the precipitate and matrix phases increases the elastic interaction and determines the precipitate shape and alignment.

In addition to the change in morphology, there is a tendency to increase the coarsening rate with the increasing lattice misfit from Figure 1 and Figure 2. A quantitative evaluation of this trend was made by measuring the average precipitate size from the total simulation area in each alloy. The statistical analysis of this information provides us with the number of nuclei, phase fraction, average radius, and dispersion of the precipitate size distribution for precipitates. The whole precipitation simulation was a continuous process, so it was easy to obtain the data at any time point.

### 3.2. Kinetic Analyses

**Figure 3:**
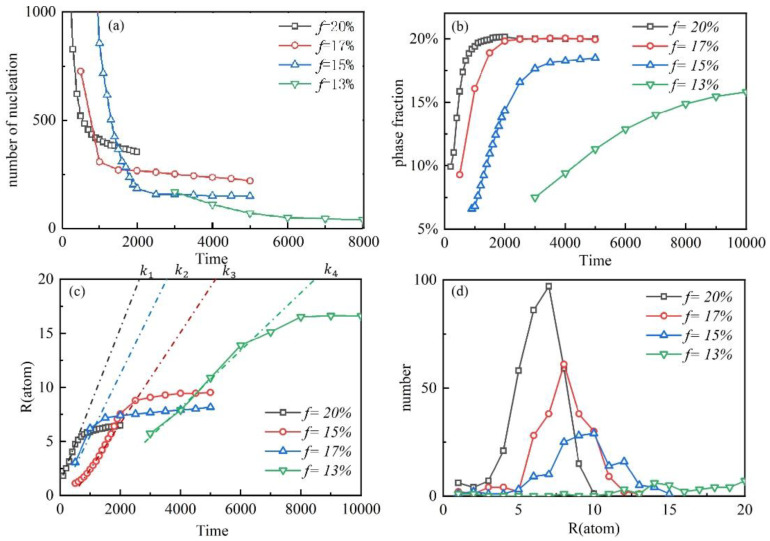
Dynamic analysis of dislocation-induced precipitation. (**a**) The number of nuclei. (**b**) The phase fractions. (**c**) The average radius of clusters. *k*_1_ = 0.0067; *k*_2_ = 0.0056; *k*_3_ = 0.0039; *k*_4_ = 0.0023. (**d**) Cluster size distribution.

Figure 3a shows the trend in the number of clusters in the process of dislocation-induced precipitation in four different misfit alloys. In general, these four alloys show similar characteristics in terms of the number of nuclei. Firstly, in the initial stage, a large number of nucleation sites are formed, which provides the basis for the formation of clusters. With the passage of time, the number of clusters gradually decreases, showing an exponential downward trend. As the dislocation-induced precipitation process proceeds, the number of clusters gradually stabilizes. This indicates that the formation and disappearance of dislocations reach an equilibrium state, and the number of clusters does not change significantly. This stable state is due to the influence of energy balance and kinetic factors in the process of dislocation-induced precipitation. In addition, the degree of lattice mismatch also affects the number of clusters. With the increase in a lattice mismatch, a large number of misfit dislocations are generated, and the increase in dislocations leads to an increase in the number of clusters, thus promoting the formation and growth of clusters.

Figure 3b shows the change of phase fraction in the process of dislocation-induced precipitation. Among them, the green light curve represents the alloy with a lattice mismatch of 13%. In the final state, the phase fraction of the alloy is about 20%. The results show that the alloy phase fraction with the highest lattice mismatch can quickly reach a stable state, and as the lattice mismatch decreases, the time required for the alloy to reach a stable state increases significantly. The change of phase fraction reflects the formation and disappearance of a phase. The alloy with a high lattice mismatch forms more dislocations due to the lattice mismatch. The formation of dislocations promotes the formation of phases, which makes the phase fraction increase rapidly and stabilize. On the contrary, alloys with smaller lattice mismatches need longer times to reach a stable state. This is because an alloy with a smaller lattice mismatch has a lower rate of dislocation formation, resulting in a slower rate of change in phase fraction. The research results can indicate that low-mismatch materials demonstrate very low kinetics, while large-mismatch go easily to a stable state. It can be emphasized here that the low-mismatch materials are in the kinetic regime, while the large-mismatch ones are thermodynamic-like.

Figure 3c shows the coarsening curve of the cluster, which describes the change of the cluster radius with the evolution time. At the initial stage of nucleation, the average radius of the cluster increases linearly, and the larger the phase fraction, the larger the slope of the linear growth. It can be seen from Figure 3b that the larger the phase fraction, the more obvious the lattice mismatch; that is, the slope of the linear growth decreases with the decrease in the lattice mismatch. When the average radius of the cluster is close to a certain threshold, the growth rate of precipitation begins to slow down until it stops growing. When the average radii of the clusters with different phase fractions are stable, it can be seen that the green curve with the smallest phase fraction represents the largest average radius of the cluster. It can be seen that the average radius of the stable cluster increases with the decrease in a lattice mismatch.

Figure 3d describes the particle size distribution of stable clusters. The most common cluster radii of the three alloys with lattice mismatches of 20%, 17%, and 15% correspond to seven, eight, and ten atoms, respectively. The mode number of the alloy with a lattice mismatch of 20% is larger than that of the two alloys with lattice mismatches of 17% and 15%, indicating that more precipitates can be produced in the alloy with a high lattice mismatch, resulting in a smaller average size. It is worth noting that the number of different cluster radii of the alloy with a lattice mismatch of 13% is almost stable at a very low level, and even some cluster radii have tended toward 0. This indicates that when the lattice mismatch of the alloy is lower than a certain value, the radii of different clusters are stable and maintain very low levels, that is, a small number of precipitates are produced.

### 3.3. The Interaction between Dislocation and Solute

**Figure 4:**
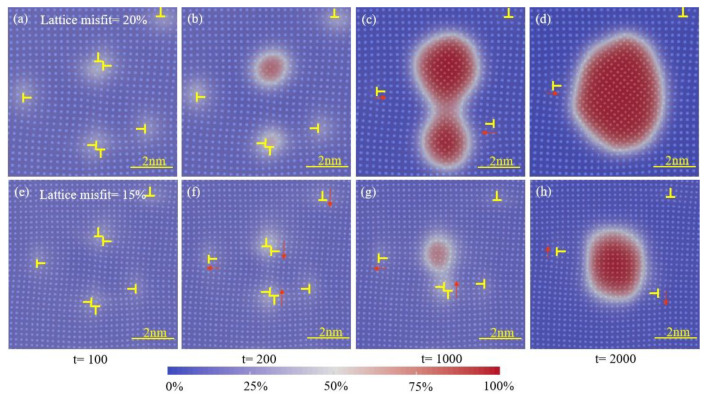
(**a**–**d**) The zoomed-in images of the area within the box labeled cluster “*a*” in Figure 1 (the color code means the concentration of the precipitation phase). (**e**–**h**) The zoomed-in images of the area within the box labeled cluster “*a*_0_” in Figure 1.

Dislocation movement and solute migration are bound to occur in the process of dislocation-induced precipitation and also affect each other. To further explore the role of lattice misfits in this process, we studied dislocation movement and solute migration under different lattice misfits during the early stage of aging. Figure 4 shows the zoomed-in images of the area within the box labeled clusters “*a*” and “*a*_0_” in Figure 1. Interestingly, these two graphs show two completely different approaches to nucleation. We observed that in alloys with a high lattice mismatch, solute atoms easily accumulate near dislocations and form precipitates due to lattice mismatches. These solute atoms will quickly aggregate at the dislocation to form two independent precipitates. As time goes on, these two precipitates will begin to coarsen and gradually increase in volume. When they contact and merge with each other, they form a larger precipitate. However, in alloys with a low lattice mismatch, it is difficult for dislocations to attract enough solute atoms for nucleation. In order to increase the amount of solute adsorption, these two dislocations will be close to each other. By approaching each other, they can share the solute atoms adsorbed on them, and these solute atoms will accumulate near the dislocations to form precipitates. This difference is caused by the degree of lattice mismatch. When the lattice mismatch is high, the stress field near the dislocation is more complex, and the solute atoms are more likely to adhere and form precipitates.

**Figure 5:**
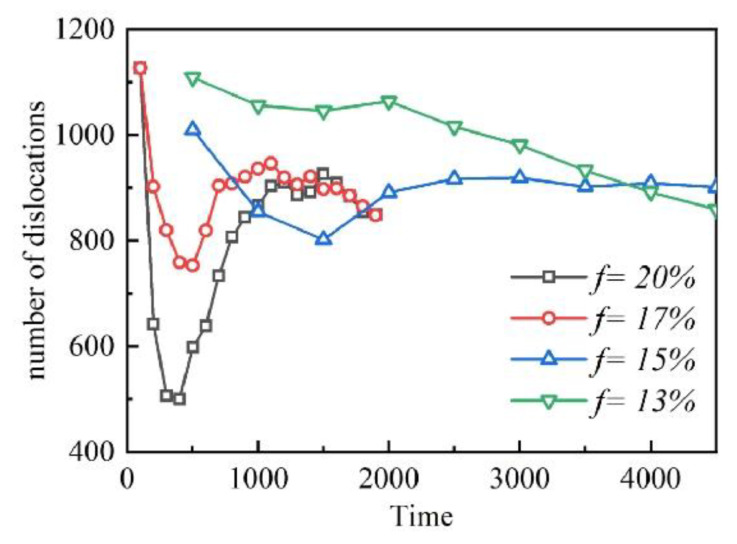
Evolution of dislocation during early aging.

In the samples with a high lattice mismatch, the number of dislocations decreases significantly in the early stage of aging and then increases gradually in Figure 5. Specifically, when there is a high lattice mismatch in the alloy, the stress field near the dislocation will be more complex. This makes it easier for solute atoms to adhere to the vicinity of dislocations, thereby forming precipitates. In the early stage of aging, the precipitates begin to coarsen and gradually increase in volume. This causes some of the smaller precipitates to dissolve and re-transform into dislocations. Therefore, the number of dislocations will decrease. However, as aging continues, the precipitates continue to coarsen and increase in volume. This makes the stress field around the dislocation more complex, and the number of solute atoms adsorbed near the dislocation increases. Therefore, the number of dislocations begins to increase. It should be noted that the increase in a lattice mismatch will only increase the high dislocation-induced precipitation rate on the basis of the alloy with a relatively high lattice mismatch. The subsequent decrease in the number of dislocations can be attributed to the precipitation of dislocations and the adsorption of dislocations in the two-phase interface.

## 4. Conclusions

In summary, we studied the influence of lattice misfits on dislocation-induced precipitation via atomic-scale analysis. Our study contributes to an atomic-scale insight into the formation of diversified precipitates in aluminum alloys in terms of lattice misfits. The main conclusions are as follows.

1. During the dislocation-induced precipitation process, the high lattice misfit can significantly increase the concentration rate of solute atoms around dislocations and induce nucleation in a short time.
2. The increasing misfit leads to a decreased critical nucleation radius, making more small precipitation continue to grow. However, the total amount of solutes is limited, and more precipitates mean a smaller size.
3. Dislocation-induced precipitation can act as pinning and hinder dislocation motion. In addition, the aggregation of dislocations can accelerate dislocation-induced precipitation. A relatively high lattice misfit will produce many precipitates during the early stage of aging, and the subsequent coarsening process will lead to the dissolution of some precipitates and the formation of dislocations.

## Data Availability

The data that support the findings of this study are available from the authors upon reasonable request.
